# ****β****-Lactamase-Producing Multidrug-Resistant Bacterial Pathogens from Tracheal Aspirates of Intensive Care Unit Patients at National Institute of Neurological and Allied Sciences, Nepal

**DOI:** 10.1155/2013/847569

**Published:** 2013-09-02

**Authors:** Santosh Khanal, Dev Raj Joshi, Dwij Raj Bhatta, Upendra Devkota, Bharat Mani Pokhrel

**Affiliations:** ^1^Central Department of Microbiology, Tribhuvan University, Kathmandu, Nepal; ^2^National College (NIST), Tribhuvan University, Kathmandu, Nepal; ^3^National Institute of Neurological and Allied Sciences (NINAS), Kathmandu, Nepal; ^4^Department of Microbiology, Institute of Medicine (IOM), Tribhuvan University, Kathmandu, Nepal

## Abstract

The widespread use of tracheal intubation and mechanical ventilation to support the critically ill patients increases the risk of development of tracheobronchitis and bronchopneumonia. This cross-sectional study was conducted with an aim to isolate and identify bacterial pathogens from tracheal aspirates producing extended-spectrum **β**-lactamase (ESBL), AmpC **β**-lactamase, and metallo-**β**-lactamase (MBL) from August 2011 to April 2012 at National Institute of Neurological and Allied Sciences (NINAS), Kathmandu, Nepal. ESBL was detected by combined disk assay using cefotaxime and cefotaxime with clavulanate, AmpC **β**-lactamase by inhibitor-based method using cefoxitin and phenylboronic acid, and MBL by Imipenem-EDTA combined disk method. 167 bacterial strains were isolated from 187 samples and majority of them were *Acinetobacter* spp. followed by *Klebsiella pneumoniae * with 32.9% and 25.1%, respectively. 68.8% of isolates were multidrug resistant (MDR) and *Acinetobacter* spp. constituted 85.4%. ESBL, AmpC **β**-lactamase, and MBL were detected in 35 (25%), 51 (37.2%), and 11 (36.7%) isolates, respectively. *Pseudomonas* spp. (42.8%) were the predominant ESBL producer while *Acinetobacter* spp. were the major AmpC **β**-lactamase producer (43.1%) and MBL producer (54.5%).

## 1. Introduction

Tracheostomy is a surgical procedure that creates an opening directly into the trachea to ventilate and aspirate the patient in critical care setting [1]. The incidence of ventilator-associated pneumonia (VAP) ranges from 10 to 25% of all intensive care unit (ICU) patients resulting in high mortality rate of 22–71%, which is 6–21 times higher in intubated patients [2]. 

The tracheostomized patients are colonized or infected with bacteria either endogenously or exogenously. Exogenous bacteria include *Pseudomonas *spp., *Acinetobacter* spp., methicillin-resistant *Staphylococcus aureus *(MRSA), and members of Enterobacteriaceae and endogenous bacteria include *Streptococcus pneumoniae*, *Haemophilus influenzae*, and* Moraxella catarrhalis*. These bacteria are usually resistant to multiple antibiotics and cause either tracheobronchitis or bronchopneumonia [3]. Risk factors for colonization or infection with multidrug-resistant bacterial species include prolonged length of hospital stay, exposure to an ICU, receipt of mechanical ventilation, colonization pressure, exposure to broad-spectrum antimicrobial agents, recent surgery, invasive procedures, and underlying severity of illness [4, 5]. 

*β*-Lactamases are the commonest cause of bacterial resistance to *β*-lactam antimicrobial agents, which are used in the treatment of various serious infections. With the increased use of antimicrobial agents, bacteria responded with a variety of new *β*-lactamases including extended-spectrum *β*-lactamases, plasmid-mediated AmpC *β*-lactamases and metallo-*β*-lactamases [6]. Infections caused by multidrug-resistant bacteria expressing *β*-lactamases pose serious challenges to clinicians because these bacteria are resistant to a broad range of *β*-lactams, including third-generation cephalosporins, and nosocomial infections caused by these organisms complicate therapy and limit treatment options [6, 7].

The emergence and spread of antimicrobial resistance due to the production of *β*-lactamases as a major problem have drawn attention to a need for better diagnostic techniques and newer drugs to allow more specific therapy. Therefore, the characterization and antibiotic susceptibility pattern of *β*-lactamase-producing organisms can lead to successful infection control, involving antimicrobial stewardship and public health interventions aimed at controlling the emergence of such life-threatening multidrug-resistant bacteria. Hence, this study was undertaken to detect the bacterial pathogens and determine the antimicrobial resistance pattern of clinically relevant bacteria producing extended-spectrum *β*-lactamase, AmpC *β*-lactamase, and metallo-*β*-lactamase from tracheal aspirate of patients admitted to ICU.

## 2. Materials and Methods

This cross-sectional study was conducted at National Institute of Neurological and Allied Sciences, Bansbari, Kathmandu, Nepal, from August 2011 to April 2012. A total of 187 tracheal aspirate samples were included in the study.

### 2.1. Specimen Collection

The samples were collected in mucus trapper by applying negative pressure through automated machine by experienced physician and samples were immediately transported to the laboratory. 

### 2.2. Culture of the Specimen

The specimens were inoculated on blood agar, MacConkey agar, and chocolate agar plates. In the chocolate agar plate, a 5 *μ*g optochin disc and a 10 U bacitracin disc were added to screen out *S. pneumoniae *and *H. influenzae*, respectively, and the plates were incubated at 37°C overnight in candle jar, whereas, the MacConkey and blood agar plates were incubated under aerobic condition [8]. 

### 2.3. Identification and Antibiotic Susceptibility Test

The isolates were identified on the basis of colony characterization, staining, and biochemical tests such as oxidase, catalase, sulfide indole motility, citrate, urea hydrolysis, triple sugar iron agar test, and coagulase tests [8]. Antibiotic sensitivity test was performed using the Kirby-Bauer disk diffusion method and sensitivity results were interpreted according to CLSI guidelines [9]. Multidrug resistance was defined as resistance to three or more of the antimicrobial agents belonging to different structural classes [10].

### 2.4. Test for ESBL, AmpC *β*-Lactamase, and MBL Production

ESBL was detected by combined disk assay using cefotaxime (30 *μ*g) and cefotaxime (30 *μ*g) with clavulanate (10 *μ*g) [9], AmpC *β*-lactamase by inhibitor-based method using cefoxitin (30 *μ*g) and cefoxitin (30 *μ*g) with phenylboronic acid (20 *μ*L) [11], and MBL by combined disk assay using Imipenem (10 *μ*g) and Imipenem (10 *μ*g) with 100 mM EDTA (10 *μ*L) [12].

## 3. Results

Out of 187 tracheal aspirate samples, 138 males and 49 females, 146 (78.1%) samples showed significant growth with 21 polymicrobial growth. 167 bacterial strains were identified and among them, 115 (68.8%) were multidrug resistant. Among 167 isolates, Gram-negative bacteria constituted 154 (92.2%) of the total isolates, among which 108 (70.1%) were MDR. Among Gram-negatives, *Acinetobacter* spp. were the most frequently isolated species with 55 (32.9%) isolates and among them, 47 (85.4%) were found to be MDR-strains. Gram-positive organisms constituted 13 (7.8%) of the total isolates and 7 (53.8%) of them were MDR. *Staphylococcus aureus *constituted 12 isolates and 6 (50%) of these were MDR. The results are shown in Table 1.

*Acinetobacter* spp. showed high rate of resistance to cefepime (96.4%), cotrimoxazole (96.4%), cefoxitin (94.5%), cefotaxime (87.3%), gentamicin (83.6%), and ciprofloxacin (80%). Similarly, high rate of resistance was observed among *K. pneumoniae* and *Pseudomonas* spp. to cotrimoxazole, cefoxitin, cefotaxime, cefepime, and ciprofloxacin. The results are shown in Table 2.

*Staphylococcus aureus* showed high rate of resistance to ampicillin (83.3%), cotrimoxazole (50%), erythromycin (41.7%), cloxacillin (33.3%), and ciprofloxacin (33.3%). The results are shown in Table 3.

ESBL production was confirmed in 35 (25%) isolates and the majority consisted of *Pseudomonas *spp. with 15 (42.8%) followed by *K. pneumoniae* with 12 (34.3%). Out of the 51 (37.2%) AmpC *β*-lactamase-positive isolates, *Acinetobacter *spp. were the most frequent ones with 22 (41.5%) followed by *K. pneumoniae* with 13 (24.5%). MBL production was confirmed in 11 (36.7%) bacterial isolates and among them, 6 (54.5%) isolates were *Acinetobacter *spp. followed by *K. pneumoniae* and *Pseudomonas *spp., each with 2 (18.2%) and a single isolate of *K. oxytoca *(9.1%). The results are shown in Table 4.

## 4. Discussion

The results of the study showed high growth rate, which was in accordance with the previous study, which reported culture positivity of 90% [13]. Polymicrobial growth was observed in one-tenth of the cases and the growth of multiple organisms from tracheal specimen has been mentioned in similar studies [14, 15]. The colonization of the oropharynx, aspiration of the contaminated secretions into the lower airway, mechanical ventilation, and endotracheal tube biofilm play important role as reservoirs for infecting microorganisms [15].

In the present study, 85.4% of *Acinetobacter* spp., the most predominant isolate of tracheal aspirate, were MDR-strains. High level of resistance by *Acinetobacter* spp. was shown against cotrimoxazole (96.4%), cefotaxime (87.3%), ciprofloxacin (80%), and amikacin (78.2%). Similar trends in antimicrobial resistance (85% to ceftazidime and ciprofloxacin, 82% to cotrimoxazole, and 67% to amikacin) of *Acinetobacter* spp. have been observed [16]. *Acinetobacter* species possess a wide array of *β*-lactamases that hydrolyze and confer resistance to penicillins, cephalosporins, and carbapenems. The other mechanisms of resistance include loss of porin proteins and presence of multiple efflux pumps that remove wide range of antibiotics out of the bacterial cell [17].

In this study, 73.8% of *K. pneumoniae* were MDR-strains. These isolates showed high level of resistance against cotrimoxazole (85.7%), cefotaxime (78.6%), gentamicin (69%), and ciprofloxacin (64.3%), which was in harmony with the previous study that reported resistance of 63.1% to cotrimoxazole, 90.5% to cefotaxime, 89% to gentamicin, and 65.8% to ciprofloxacin [18]. High level of drug resistance seen among *K. pneumoniae* is mediated by the production of various types of *β*-lactamases primarily ESBL, AmpC, and metallo-*β*-lactamases along with drug efflux [19].

In the present study, 51.3% of *Pseudomonas* spp. were MDR-strains. *Pseudomonas* spp. were resistant to cotrimoxazole (86%), cefotaxime (83.8%), ciprofloxacin (51.4%), and gentamicin (43.2%) which was comparable to the results of two studies [18, 20]. *Pseudomonas *spp. display an elevated level of drug resistance mechanisms that include production of different types of *β*-lactamases primarily ESBL, AmpC enzymes, and metallo-carbapenemases, aminoglycoside-modifying enzymes, loss of porin proteins, and the presence of efflux pumps like MexAB-Opr M [17].

ESBL production was confirmed in 35 (25%) screen-positive isolates and the highest number of ESBL production was detected in *Pseudomonas* spp. (42.8%) followed by *K. pneumoniae* (34.3%), which was in contrary to one of the studies conducted in Nepal that showed higher prevalence of *E. coli* with 80% and *K. pneumoniae *with 57.1% [21]. Higher rate of ESBL production in *P. aeruginosa* has now been increasingly reported due to predominantly occurring SHV- and OXA-type ESBLs [7].

AmpC *β*-lactamase was confirmed in 51 (37.2%) of the screen-positive isolates and *Acinetobacter* spp. constituted 22 (43.1%) followed by *K. pneumoniae* with 13 (25.5%). Plasmidic AmpC genes are derived from the chromosomal AmpC genes of *Enterobacter cloacae*, *Citrobacter freundii*, *Morganella morganii*, and *Hafnia alvei*. Most plasmid-mediated AmpC *β*-lactamases are constitutively expressed, but some enzymes, such as DHA-1, DHA-2, ACT-1, CFE-1, and CMY-13, are inducible and may be more clinically dangerous conferring the capability for an organism to become more resistant during *β*-lactam therapy [22]. In this study, phenylboronic acid was used as an inhibitor of AmpC *β*-lactamase and high sensitivity and specificity of 90% and 98.2%, respectively, for this method have been reported [11].

MBL production was confirmed in 11 (36.7%) of the screen-positive isolates that constitute 54.5% of *Acinetobacter *spp., *K. pneumoniae*, and *Pseudomonas *spp., each with 18.2% and 9.1% of *K. oxytoca.* In contrast with this finding, a Korean study reported MBL production in only 14.2% of *A. baumannii* and 11.4% of *P. aeruginosa* [23]. The most common transferable MBL families include the VIM-, IMP-, GIM-, SPM-, and SIM-type enzymes, which have been detected primarily in *P. aeruginosa* but are also found in other Gram-negative bacteria, including nonfermenters and members of the family Enterobacteriaceae [24]. MBL-producing bacteria are an increasing public health problem worldwide and mortality rates have been increased due to inadequate empirical therapy [25]. In the present study, MBL production was detected using Imipenem-EDTA combined disk method, which has sensitivity and specificity of 100% and 98%, respectively [26].

## 5. Conclusion

We conclude that Gram-negative bacilli were the predominant isolates of tracheal aspirate of ICU patients. There is a high rate of resistance to cephalosporins, fluoroquinolones, aminoglycosides, and cotrimoxazole. *β*-lactamases confer a high level of resistance to *β*-lactam antibiotics and these traits are usually carried in transferable genes, which are capable of being acquired by normally nonpathogenic bacteria. Therefore, early detection in routine laboratory, immediate infection control, and antibiotic stewardship programs should be implemented in order to limit the spread of *β*-lactamase-producing organisms.

## Figures and Tables

**Table 1:** Pattern of microbial isolates from tracheal aspirate of ICU patients.

Organisms	Frequency	MDR (%)
Gram-negative bacteria	**154**	**108 (70.1)**
* Acinetobacter *spp.	55	47 (85.4)
* K. pneumoniae *	42	31 (73.8)
* Pseudomonas* spp.	37	19 (51.3)
* E. coli *	12	6 (50)
* Enterobacter *spp.	3	2 (66.7)
* K. oxytoca *	2	2 (100)
* Citrobacter freundii *	2	0
* Proteus vulgaris *	1	1 (100)
Gram-positive bacteria	**13**	**7 (53.8)**
* S. aureus *	12	6 (50)
* S. pneumoniae *	1	1 (100)
**Total**	**167**	**115 (68.8)**

**Table 2:** Antibiotic resistance rates (%) for predominant Gram-negative bacilli recovered from tracheal aspirate of ICU patients.

Antibiotics	*Acinetobacter *spp. (*N* = 55)	*K. pneumoniae *(*N* = 42)	*Pseudomonas *spp. (*N* = 37)	*E. coli *(*N* = 12)
Amikacin	78.2	54.8	40.5	8.3
Ampicillin	NT	100	NT	100
Carbenicillin	NT	NT	43.2	NT
Cefepime	96.4	83.3	78.4	66.7
Cefotaxime	87.3	78.6	83.8	58.3
Cefoxitin	94.5	81	89.2	66.7
Ciprofloxacin	80	64.3	51.4	66.7
Cotrimoxazole	96.4	85.7	86.5	83.3
Erythromycin	NT	NT	NT	25
Gentamicin	83.6	69	43.2	25
Imipenem	23.6	9.5	16.2	0
Ofloxacin	80	64.3	51.4	66.7
Piperacillin-tazobactam	69.1	57.1	29.7	NT
Polymyxin B	0	0	0	NT

*NT: not tested.

**Table 3:** Antibiotic resistance rates (%) for Gram-positive cocci recovered from tracheal aspirate of ICU patients.

Antibiotics	*S. aureus *(*N* = 12)	*S. pneumoniae *(*N* = 1)
Amikacin	NT	100
Ampicillin	83.3	100
Cefoxitin	33.3	100
Cefotaxime	NT	100
Cloxacillin	33.3	NT
Ciprofloxacin	33.3	0
Cotrimoxazole	50	100
Erythromycin	41.7	100
Gentamicin	33.3	100
Methicillin	33.3	NT
Vancomycin	0	0

*NT: not tested.

**Table 4:** Profile of *β*-lactamase-producing bacterial strains from tracheal aspirate of ICU patients.

Organisms	ESBL producers (no. and %)	AmpC *β*-lactamase producers (no. and %)	MBL producers (no. and %)
*Acinetobacter *spp.	5 (14.3)	22 (43.1)	6 (54.5)
*K. pneumoniae *	12 (34.3)	13 (24.5)	2 (18.2)
*Pseudomonas *spp.	15 (42.8)	9 (17.6)	2 (18.2)
*E. coli *	3 (8.6)	4 (7.8)	0
*Enterobacter *spp.	0	2 (4)	0
*K. oxytoca *	0	1 (2)	1 (9.1)
*C. freundii *	0	0	0
*P. vulgaris *	0	0	0
*S. aureus *	0	0	0
*S. pneumoniae *	0	0	0
**Total**	**35 (25) **	**51 (37.2)**	**11 (36.7)**
